# Multiphoton FLIM imaging of NAD(P)H and FAD with one excitation wavelength

**DOI:** 10.1117/1.JBO.25.1.014510

**Published:** 2020-01-09

**Authors:** Ruofan Cao, Horst Wallrabe, Ammasi Periasamy

**Affiliations:** a University of Virginia, WM Keck Center for Cellular Imaging, Department of Biology, Charlottesville, Virginia, United States; b University of Virginia, Department of Biomedical Engineering, Charlottesville, Virginia, United States

**Keywords:** NADH, FAD, FLIRR, FLIM, metabolic imaging

## Abstract

Two-photon fluorescence lifetime imaging microscopy (FLIM) is widely used to capture autofluorescence signals from cellular components to investigate dynamic physiological changes in live cells and tissues. Among these intrinsic fluorophores, nicotinamide adenine dinucleotide (phosphate) (NAD(P)H) and flavin adenine dinucleotide (FAD)—essential coenzymes in cellular respiration—have been used as intrinsic fluorescent biomarkers for metabolic states in cancer and other pathologies. Traditional FLIM imaging for NAD(P)H, FAD, and in particular fluorescence lifetime redox ratio (FLIRR) requires a sequential multiwavelength excitation to avoid spectral bleed-through (SBT). This sequential imaging complicates image acquisition, may introduce motion artifacts, and reduce temporal resolution. Testing several two-photon excitation wavelengths in combination with optimized emission filters, we have proved a FLIM imaging protocol, allowing simultaneous image acquisition with a single 800-nm wavelength excitation for NADH and FAD with negligible SBT. As a first step, standard NADH and FAD single and mixed solutions were tested that mimic biological sample conditions. After these optimization steps, the assay was applied to two prostate cancer live cell lines: African-American (AA) and Caucasian-American (LNCaP), used in our previous publications. FLIRR result shows that, in cells, the 800-nm two-photon excitation wavelength is suitable for NADH and FAD FLIM imaging with negligible SBT. While NAD(P)H signals are decreased, sufficient photons are present for accurate lifetime fitting and FAD signals are measurably increased at lower laser power, compared with the common 890-nm excitation conditions. This single wavelength excitation allows a simplification of NADH and FAD FLIM imaging data analysis, decreasing the total imaging time. It also avoids motion artifacts and increases temporal resolution. This simplified assay will also make it more suitable to be applied in a clinical setting.

## 1 Introduction

Fluorescence lifetime imaging microscopy (FLIM) is applied in broad areas of the life sciences and industrial fields for its ability to capture information from a smaller focal volume, inter alia being independent of fluorophore concentration, but sensitive to environmental changes such as pH and temperature and other advantages, which can all be exploited in scientific investigations.^1^^–^^9^ Fluorescence lifetime is of particular interest for quantitative studies in scattering and absorbing samples, such as tissue sections, where intensity-based methods are problematic.^10^^–^^14^ When FLIM is combined with multiphoton (MP) excitation, greater focal depth is achieved, important for thicker tissue specimens, and out-of-focus fluorescence is avoided with a smaller focal volume, without the need for a confocal pinhole. Two FLIM methods are available: frequency-domain FLIM and time-domain FLIM.^15^^–^^20^ This paper uses the latter, also called time-correlated single-photon counting (TCSPC).^21^ MP excitation illuminates molecules by infrared ranges that would otherwise require single-photon excitation in the UV region, generally undesirable to live cells, because of phototoxicity at longer exposure.^22^

Beginning with the seminal work by Chance in the 1960s,^23^^,^^24^ exploiting the autofluorescent properties of the coenzymes NADH (reduced form of nonfluorescent NAD+) and flavin adenine dinucleotide (FAD) (oxidized form of nonfluorescent FADH2) for measuring the cellular reduction/oxidation (REDOX) states noninvasively, the REDOX field has grown exponentially.^25^^,^^26^ First, intensity-based methods were expanded,^27^^–^^31^ followed by fluorescence lifetime imaging assays to monitor REDOX changes as markers for changed metabolic states.^12^^,^^30^^,^^32^^–^^37^ These assays are of particular interest in measuring responses to treatment in various cancer pathologies, cancer being a metabolically heterogeneous pathology, being able to generate energy by oxidative phosphorylation (OXPHOS) and (often preferentially) by glycolysis.^9^^,^^38^^–^^41^ A third coenzyme, nicotinamide adenine dinucleotide (phosphate) [NAD(P)H], the phosphorylated form of NADH, cannot spectrally be differentiated from NADH, so this paper follows convention to describe the mixed lifetime/intensity signal as NAD(P)H. A heightened interest in expanding the application and simplification of this FLIM assay arises from its potential to test suitability and earliest response to drug treatment in cancer therapies. Chemotherapy response in animal models and patients typically take days or weeks; this *in vitro* assay has been shown to predict results in hours,^35^ potentially helpful to devise more individualized treatment modalities for patients.

While analysis of several FLIM parameters provides insights into physiological events in cells and tissues for these intrinsically fluorescent coenzymes, the key indicators are described in Table 1. The cascade of reactions involving several enzymes, changing NAD+/NADH, NADP+/NADH, and FADH2/FAD ratios and the NAD(P)H/FAD intensity REDOX ratio,^42^^–^^44^ as well as the recently suggested preferred REDOX ratio measurement, fluorescence lifetime redox ratio (FLIRR),^34^ can be tracked by these FLIM parameters. The intensity fractions a1 and a2 are the pre-exponential parameters associated with the shorter (τ1) and longer (τ2) lifetime components of a biexponential fluorescence decay model. These parameters are determined by fitting the model to the measured fluorescence decay data on a per pixel basis. a1% and a2% are normalized parameters according to a1%=a1/(a1+a2) and a2%=a2/(a1+a2).

**Table 1:** Main FLIM parameters.

Parameters	Names
NAD(P)H-τ1	Free, nonenzyme-bound lifetime
NAD(P)H-a1%	Free, nonenzyme-bound fraction
FAD-τ2	Free, nonenzyme-bound lifetime
FAD-a2%	Free, unquenched fraction
NAD(P)H-τm^a^	Average lifetimes
NAD(P)H-τ2	Enzyme-bound lifetime
NAD(P)H-a2%	Enzyme-bound fraction
FAD-τ1	Enzyme-bound lifetime
FAD-a1%	Quenched fraction
FAD-τm	Average lifetime

a τm=(τ1×a1%)+(τ2×a2%)/(a1%+a2%).

Technological advances in FLIM instrumentation (hybrid detectors and lasers) and software developments have greatly optimized data acquisition and sensitivity of FLIM output. Yet, mostly to avoid spectral bleed-through (SBT), the acquisition of NAD(P)H and FAD lifetime signals is commonly executed sequentially by illuminating the former in the 700- to 740-nm range and the latter in the 880- to 900-nm range.^45^^–^^50^ This sequential imaging complicates image acquisition and may introduce motion artifacts and reduce temporal resolution. Some investigators have applied an expensive solution to the problem, using two parallel lasers, cutting down the laser wavelength switch-over time.^51^^,^^52^ A one-excitation wavelength has been proposed^53^ to excite both NAD(P)H and FAD at the same time. However, there is a need for a comprehensive study to cover aspects of intensity spectrum and fluorescence lifetime.

This paper presents an assay, using one wavelength (800 nm) to acquire NAD(P)H and FAD FLIM signals simultaneously with optimized emission filters. We observed negligible SBT, tested first investigating single solutions of both coenzymes, followed by mixed solutions to mimic cellular conditions, and finally applied the assay to two prostate cancer (PCa) cell lines, this group has used in previous publications.^33^^–^^35^ We compared the intensity spectrum and fluorescence lifetime results with other wavelengths and finally found the optimized excitation wavelength. The data are compared with the common standard assay of sequentially exciting cells with 740 nm followed by 890 nm at the new filter combination.

## 2 Materials and Methods

### 2.1 Cell Culture

Different PCa cell lines from African-American (E006AA; provided by Roswell Park Cancer Institute) and Caucasian (LNCaP) origins have been used in this study. The E006AA (or AA) cells were maintained in high-glucose Dulbecco’s modified Eagle medium (Life Technologies) supplemented with 10% cosmic calf serum (Hyclone), 1% penicillin–streptomycin (Life Technologies), and 4 mM sodium pyruvate (Life Technologies). The LNCaP cells were maintained in RPMI 1640 (Life Technologies) supplemented with 10% cosmic calf serum (Hyclone) and 1% penicillin–streptomycin (Life Technologies). All cells were maintained in the cell culture incubator, at 37°C with 5% ${\mathrm{CO}}_{2}$.

### 2.2 Instrumentation

A Zeiss LSM-780 NLO confocal/MP microscopy system consists of an inverted Axio Observer (Zeiss) microscope, motorized stage for automated scanning, Chameleon Vision-II (Coherent Inc.) ultrafast Ti:sapphire laser with dispersion compensation to maintain pulses at the specimen plane (690 to 1060 nm, 80 MHz, 150 fs) for MP excitation, and a standard set of dry and immersion objectives. Two HPM-100-40 hybrid GaAsP detectors (Becker and Hickl) are coupled to the nondescanned port of the microscope using two T-adapters (Zeiss) with proper dichroics and bandpass filters to collect as much fluorescence as possible in the spectral ranges *Em*, NAD(P)H channel: 450/50 nm FAD channel: 560/80 nm. The two channels also contain a 690-nm shortpass filter (Zeiss) in the beam path to avoid excitation background. Two SPC-150 cards (Becker and Hickl) synchronized with the pulsed laser and the Zeiss LSM-780 scan head signals collect the time-resolved fluorescence in TCSPC mode using SPCM (version 9.74) acquisition software. A motorized stage is used, an adjustable mini incubator maintains the temperature of specimens at 37°C under humidified blood–gas mixture conditions during imaging using Zeiss 40× NA1.3 oil apochromatic objective lens.

### 2.3 NADH, FAD and Calibrated Solutions

NADH (Roche 10107735001) and FAD (Sigma F6625) are diluted in 1× PBS (Gibco 10010023) to the final concentration of 150 and 100  μM, respectively. The calibrated solution has the mixture of the NADH and FAD at same concentrations.

### 2.4 Emission Spectrum Imaging

The emission spectra are collected under the lambda mode of Zeiss LSM-780 NLO. The emission range is set from 415 to 615 nm and GaAsP detector set at a gain of 700.

### 2.5 FLIM Imaging

For solutions, excitation scanning was executed from 720 to 890 nm with 20-nm intervals. The power of the laser was kept the same (average power 7 mW) during the scanning. Each wavelength uses 60-s collection time. FLIM images for cell specimens were collected under the same condition as the solutions.

For the drug treatment experiment, we recorded several field-of-view (FOV) positions at the before-treatment control image acquisition. PCa cells were then treated on microscope stage with 1-μM doxorubicin and reimaged at the same FOVs at 20, 40, 60, 80, 100, and 120 min after treatment. Acquisition of NAD(P)H and FAD channel images simultaneously at 800 nm was followed immediately by the traditional imaging method [NAD(P)H at 740 nm FAD at 890 nm], both at 60 s (average power 7 mW at the specimen plane) for each wavelength.

### 2.6 FLIM Fitting

SPCImage 8.0 was used for FLIM fitting. Fitting conditions are established based on the B&H handbook; 1-component incomplete model was used to fit pure NADH and FAD solutions, and 2-component incomplete model to fit calibrated solutions and cellular images. The offset and scattering are set to “0.” Shift is optimized by testing several pixel positions on morphologies of interest for optimal $\chi^{2}$ as close to 1 and shift is then fixed. These are preferred settings for fitting 2p-FLIM images with hybrid detectors and taken under complete black-out conditions.

### 2.7 FLIM Processing and Analysis

**Fig. 1:**
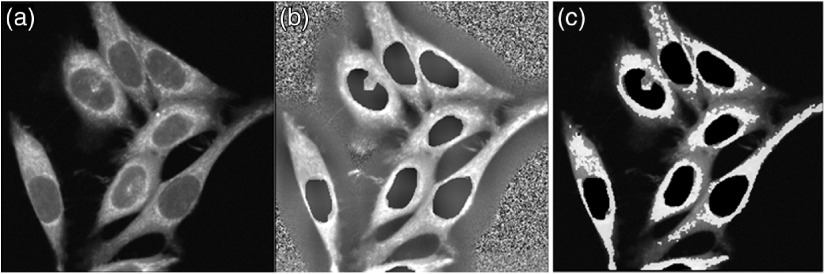
Cell normalization and ROI selection in HeLa cells: (a) NAD(P)H intensity was used to isolate dominant mitochondrial morphology. (b) After zeroing the nuclear region, intensity images were normalized to compensate for varying intensities in a time course. (c) Pixel ROIs are generated by thresholding to ranges isolating mitochondrial morphology.

The FLIM processing follows as previous published.^33^ In short, photon reference images are normalized [Figs. 1(a)–1(b)] to compensate for varying intensities (FIJI, plugins → integral image filters → normalize local contrast): followed by zeroing the nucleus and creating single pixel region-of-interest (ROI) by an ImageJ/FIJI custom plugin. The purpose of this sequence is to create pixel locations by X−Y coordinates, specific for mitochondrial [Fig. 1(c)] morphologies. Those locations are then applied to the FLIM data to extract FLIM parameters in the data pool. A custom Python code ultimately analyzes different data combinations to produce ratios, means, medians, and histograms, further charted in MS Excel. FLIRR images are generated using MATLAB.

## 3 Results

### 3.1 Testing Single Solutions of NADH and FAD

**Fig. 2:**
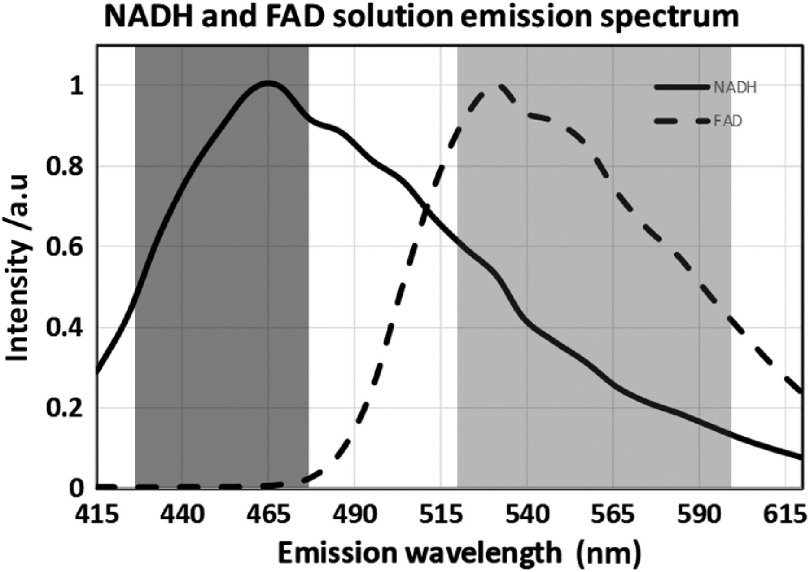
Emission spectrum of single NADH (150  μM) and FAD (100  μM) in solution. Emission spectrum of NADH (solid line) and FAD (dashed line). Optimized filters for NADH (dark gray band, 450/50 nm BP) and FAD (light gray band, 560/80 nm BP).

**Fig. 3:**
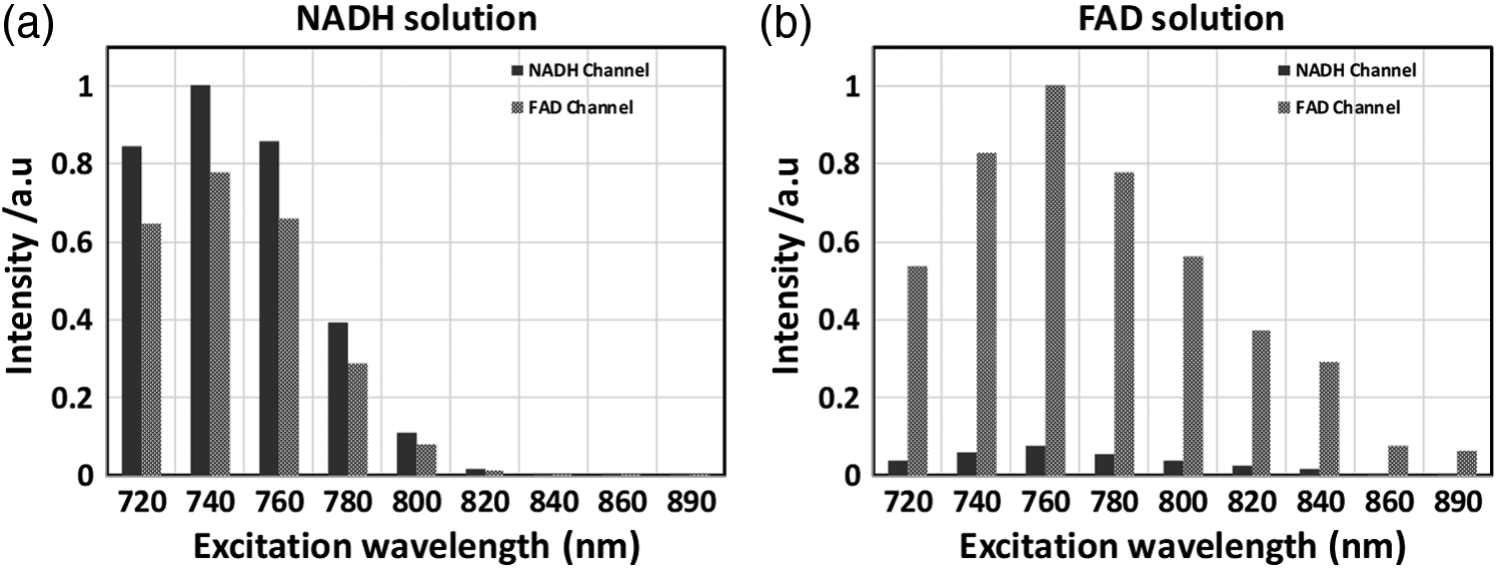
Normalized intensity levels measured at a range of two-photon excitation wavelengths (720 to 890 nm at 20-nm intervals): (a) NADH solution, 150  μM, showing normalized intensity levels in the NADH and FAD channels at indicated excitation wavelengths on the x-scale. (b) FAD solution, 100  μM, showing normalized intensity levels in the NADH and FAD channels at indicated excitation wavelengths on the x-scale. At the excitation range 780 to 800 nm, the NADH SBT into the FAD channel is leveling off, and likewise, FAD back-SBT into NADH channel is very low.

Based on published experiments^54^^,^^55^ of NADH and FAD in solution, we optimized the concentrations of the two coenzymes to 150  μM for NADH and to 100  μM for FAD to match cell intensities. Several related objectives determined the choice of the final emission filter selection of NADH at 450/50 nm and FAD at 560/80 nm (Fig. 2) and optimal excitation wavelength: (a) minimizing NADH SBT into the FAD channel and (b) avoiding back-SBT of FAD into the NADH channel. The first objective can be met as Fig. 3(a) demonstrates: NADH signal and its SBT into the FAD channel are leveling off dramatically from 740- to 820-nm excitation, still resulting in sufficient photon count at 800 nm in the NADH channel for correct fitting. This shows a potential excitation wavelength point where the FAD signal will be dominant in the FAD channel. The percentage of the NADH bleed-through will be illustrated in the later fluorescence lifetime experiments. The second objective is met showing negligible FAD SBT in the NADH channel [Fig. 3(b)]. Here, the two solutions were excited at a 2% power level (∼7  mW) throughout the 720 to 890 range of wavelengths at 20-nm intervals. The final interval was 30 nm to show the traditional excitation wavelength for FAD. The 890-nm traditional excitation wavelength of FAD does not generate a bright signal [800 provides a 10-fold photon increase over 890 nm as shown in Fig. 3(b)], the main reason why a higher laser power is required at 890 nm. Our proposed 800-nm excitation wavelength generates a larger signal than at 890 nm at the same power level as NADH excitation.

### 3.2 Intensities at Different Excitation Wavelengths—Mixed NADH and FAD Calibration Solutions, Plus 3 Different Cell Lines

**Fig. 4:**
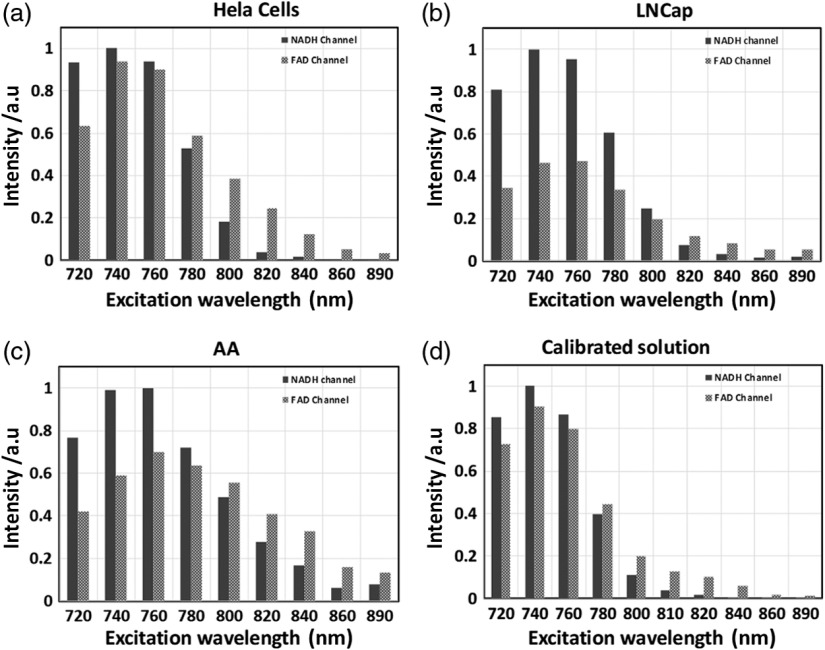
Normalized intensity levels measured at a range of two-photon excitation wavelengths (720 to 890 nm at 20-nm intervals). (a–c) HeLa, African-American/Caucasian LNCaP PCa cells at normalized intensity levels in the NADH and FAD channels at indicated excitation wavelengths on the x-scale. (d) Calibrated solution, a mix of NADH (150  μM) and FAD (100  μM), at normalized intensity levels in the NADH and FAD channels at indicated excitation wavelengths on the x-scale. Compared with single solutions, cells are subject to other variables (e.g., REDOX states, OXPHOS versus glycolysis balance), but generally follow the same trends in Fig. 4. The calibrated solution can therefore serve to mimic cellular conditions.

Following the experimentation with single solutions of NADH and FAD, the same approach was used in a mixed solution of the two coenzymes at same molar concentrations, named “calibrated solution.” In addition, three cell lines (HeLa human cervical cancer cells, AA African, and Caucasian LNCaP PCa cells) were also excited at 20-nm intervals from 720 to 890 nm and their intensities recorded in the NADH and FAD channels, emission filters 450/50 and 560/80 nm, respectively (Fig. 4). At the different wavelength points in the FAD channel, the calibrated solution predicts a mixture of NADH SBT and FAD intensities, being highest at 740 to 760 nm. The three cell lines follow similar trends and show expected intercell line variability, influenced by the REDOX states and OXPHOS versus glycolysis ratios. The calibrated solution serves therefore as a good approximation to mimic cellular conditions.

### 3.3 Emission Spectrum of Calibration Solution at Different Two Photon Excitation Wavelengths

**Fig. 5:**
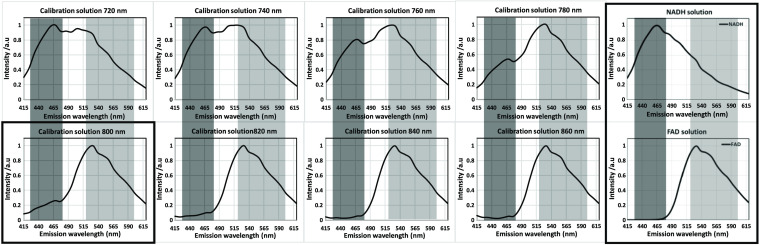
Emission spectrum of calibration solution (150  μM NADH+100  μM FAD) at different two-photon excitation wavelength. NADH emission filter 450/50 BP (dark gray band) and FAD 560/80 (light gray band). Calibration solution was excited from 720 to 860 nm in 20-nm steps. At 800 nm, a “sweet spot” becomes apparent, with virtually no back-SBT from FAD into the NADH channel and FAD emission of the calibration solution matching that of the single FAD solution, indicating negligible or no SBT contribution into the FAD channel (framed boxes, on the right and at the bottom left). While photon counts for NADH are visibly reduced, they are sufficient for FLIM fitting.

Having established that the calibration solution was a good approximation and standard for measuring the effects of changing the excitation wavelength, this time the emission spectra at each excitation point were recorded (720 to 860 nm in 20-nm steps) (Fig. 5). At 800 nm, a “sweet spot” becomes apparent, with virtually no back-SBT from FAD into the NADH channel and negligible SBT of NADH into the FAD channel with acceptable NADH intensity in the NADH channel, as predicted in Figs. 3 and 4. Both calibration solution results are compared with the single solutions spectra and found to be matching (red boxes in Fig. 5). Looking at alternative single-excitation wavelengths, there is an increased NADH signal at 780 nm compared to 800 nm, but still some spectral SBT into the FAD channel. At 820 nm, the spectra are almost the same as pure FAD solution, but the NADH signal becomes too low for accurate lifetime fitting, leaving the 800-nm excitation as the most balanced option, even though, photon counts for NADH are visibly reduced, while sufficient for FLIM fitting.

### 3.4 Fluorescence Lifetime of Single NADH and FAD Solutions versus Calibration Solution

**Fig. 6:**
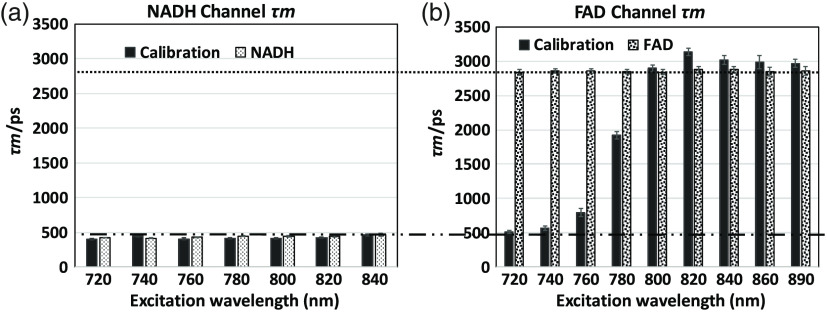
Average (τm) fluorescence lifetime results of single NADH and FAD solutions versus calibration solution at different two-photon excitation wavelengths: (a) NADH emission channel. In the absence of any meaningful FAD back-SBT, both, mixed calibration and NADH solutions show only the NADH lifetime values at one-component fitting. (b) FAD emission channel: here, the calibration solution is at two-component fitting, because of NADH-SBT; here τm consists of τ1 NADH lifetime and τ2 FAD lifetime. At 720- and 740-nm excitation, τm is almost equal to the NADH lifetime, indicating that the majority of the signal is contributed by the NADH-SBT. At 760 and 780 nm, increasingly the FAD fraction dominates τm as the NADPH-SBT fraction declines. At 800 nm, the FAD lifetime matches that of the single FAD solution with virtually no contribution from NADH SBT, an important observation for supporting the choice of this wavelength for both coenzymes.

The effects on lifetime at different excitation wavelengths, described earlier, are shown in Fig. 6. NADH has a single exponential decay of 500 ps while FAD has a single exponential decay of 2800 ps. In the NADH channel, the excitation only extends to 840 nm because after 840 nm, there are insufficient photons for a good fitting (see Fig. 3). Consequently, the single solutions of NADH and FAD are fitted mono-exponentially in their respected emission channels; this also applies to the calibration solution in the NADH channel, where no detectable FAD back-SBT occurs and only NADH signal is detected. The fitting of the mixed calibration solutions is more complex in the FAD channel where the detected signal contains both FAD and NADH bleed-through; when the pure FAD signal is contaminated with NADH SBT, a biexponential fitting is appropriate and τm (see Table 1) is used for comparison. In this fitting condition, the τ1 is the NADH lifetime and τ2 is the FAD lifetime. The lifetime of the calibration solution from 720- to 740-nm excitation is mostly NADH SBT (a1%=∼100%), matching the lifetime in the NADH channel. At 760/780 nm, the rising lifetime shows an increasing share of FAD contribution and diminishing NADH SBT contamination. At 760 nm, τm=∼800  ps; based on Table 1 equation τm=τ1 a1%+τ2a2%, we can calculate a1%, the intensity fraction of NADH, which is ∼86%, and a2%=1−a1%=14%. At 780 nm, τm=∼2000  ps, a1%=35%, a2%=65%. After 800 nm, a “pure” FAD signal (a2%=∼100%) appears, as indicated by the matching level of the single FAD solution [Fig. 6(b)]. From 800 to 890 nm, we know the FAD signal drops dramatically, so we finally picked 800 nm as our optimized wavelength to collect the best signal with negligible NADH bleed-through. The greater lifetime is probably due to the dual-model fitting in calibration solution compared to the single-model fitting used in the pure solution. In short, this data support the choice of the single wavelength excitation at 800 nm.

### 3.5 FLIM Parameters of NAD(P)H and FAD at Different Excitation Wavelengths in Two Cell Lines

**Fig. 7:**
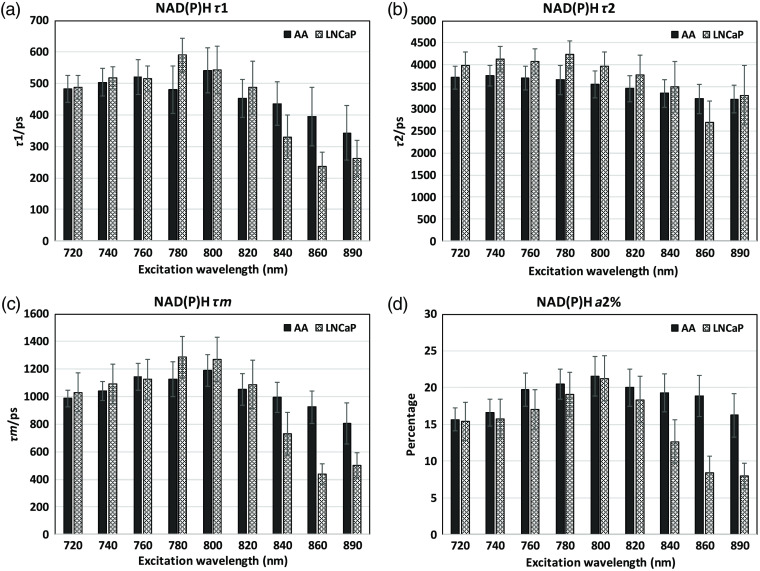
Fluorescence lifetime parameter results at different excitation wavelengths in the NAD(P)H channel for AA and LNCaP PCa cells: mean data for AA-African-American PCa cells (solid bars) and Caucasian LNCaP PCa cells (light, patterned bars). (a) NAD(P)H-τ1, (b) NAD(P)H-τ2, (c) NAD(P)H-τm, (d) NAD(P)H-a2%. We suggest that after 800 nm, the photon count is too low for accurate fitting.

**Fig. 8:**
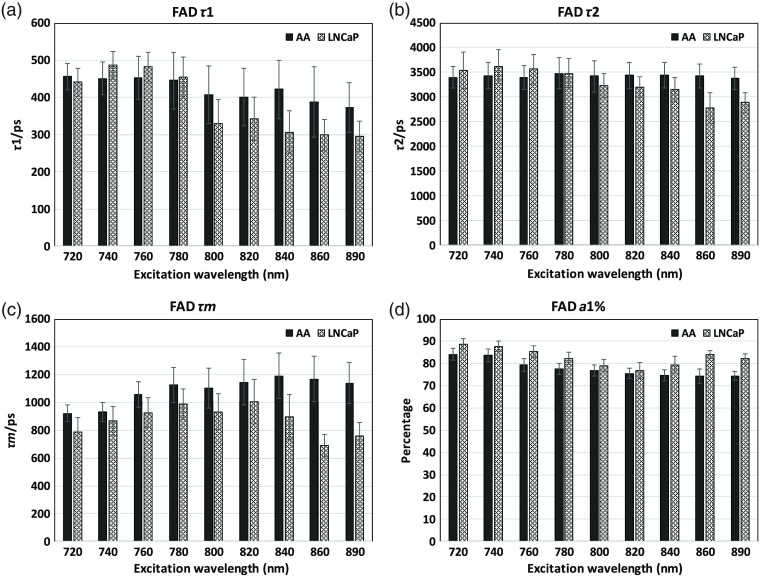
Fluorescence lifetime parameter results at different excitation wavelengths in the FAD channel for AA and LNCaP PCa cells. Mean data for AA-African-American PCa cells (solid bars) and Caucasian LNCaP PCa cells (light, patterned bars): (a) FAD-τ1, (a) FAD-τ2, (c) FAD-τm, and (d) FAD-a1%.

In Figs. 7 and 8, FLIM parameter results for τ1, τ2, τm, and a2% at range of wavelengths are described in African-American and Caucasian PCa cell lines, focusing on how the chosen single 800-nm excitation wavelength compares with the common 740 nm for NAD(P)H and 890 nm for FAD. We show that there are some absolute differences compared to solutions because the NAD(P)H and FAD in cells exist in both free and bound types. All the results are fitted in double exponential decays. For NAD(P)H-τ1, the 800-nm excitation results are similar to the 740-nm NAD(P)H results, after 800 nm, the value decreases due to the considerable photon drop (Fig. 4) which causes bad fitting. For NAD(P)H a2%, the result at 800 excitation is larger than at 740 excitation, probably due to the change of quantum yield of NAD(P)H-bound and free moieties. In summary, compared to the 740-nm excitation, 800 delivers similar τ1 and τ2 values, confirming the rationale of using 800 nm for NAD(P)H excitation. NAD(P)H-τm’s differences are mainly driven by τ1 and a2%% (see equation in Table 1).

For FAD (Fig. 8), the situation is similar, comparing the chosen 800-nm excitation wavelength with the traditional 890 nm. The 800-nm excitation shows similar results as 890 nm in τ1,τ2 values. For FAD-τ1, before 800 nm, the values increase to the NAD(P)H-τ1 range indicating the NAD(P)H-SBT. FAD a1% changes before 800 nm is mainly due to the NAD(P)H-SBT and the changes after 800 nm are suggested to be due to the change of quantum yield of FAD-bound and free moieties. The FAD τm’s differences are mainly driven by τ1 and a1%.

In summary, the 800-nm excitation generates essentially the same NAD(P)H and FAD τ1, τ2 results compared to the traditional excitation wavelengths. At 800-nm excitation, both NAD(P)H and FAD are excited at the same time, decreasing the complexity and time of imaging and increasing the temporal resolution. The NAD(P)H-a2% and FAD-a1% have some differences in their absolute levels at 800 nm because of the different quantum yields of bound and free species. This difference does not diminish the utility of 800 nm because the absolute values of relative fractions are less meaningful in the presence of interventions where the changes of relative fractions reflect the metabolic shifts. We tested drug responses and show changes of these fractions under the next heading.

### 3.6 LNCaP Prostate Cancer Cells Treated with Doxorubicin—Comparing 800-nm versus 740/890-nm Excitation

We previously developed the FLIRR measurement assay, initially driven by the need to make this measurement in tissue sections, where the common intensity-based REDOX ratio is unsuitable because of light scattering and other intensity artifacts.^34^ We named this assay FLIRR for fluorescence lifetime redox ratio, which consists of a ratio of the enzyme-bound fractions, i.e., NAD(P)H-a2%/FAD-a1%.^34^ As reported previously, this ratio increases after doxorubicin treatment, the putative mechanism being a normalization of OXPHOS, and in addition, restoring the ROS equilibrium, which is disturbed as one of cancer’s strategies to block the apoptosis pathway. The single wavelength assay is particularly relevant for the FLIRR measurement avoiding motion and temporal artifacts potentially present in sequential imaging.

**Fig. 9:**
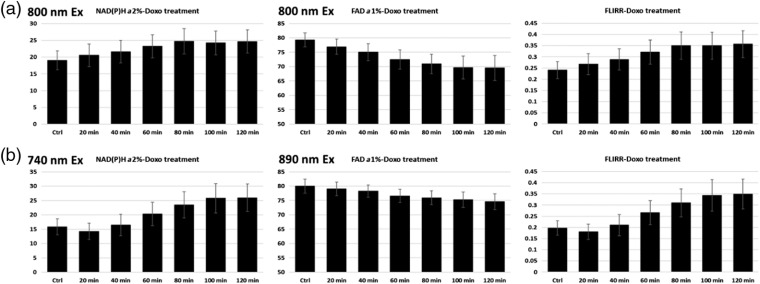
Comparison of FLIRR parameters NAD(P)H-a2% and FAD-a1% and their ratio at 800 nm versus 740/890-nm excitation in LNCaP PCa cells. (a) All data are based on single-excitation wavelength 800 nm for NAD(P)H and FAD. FLIRR is the ratio of NAD(P)H-a2%/FAD-a1%, a marker for cellular REDOX. (b) NAD(P)H-a2% based on 740-nm excitation, FAD-1% at 890-nm excitation and FLIRR as described above. Data from both wavelength approaches are from identical FOVs and pixel locations. While some differences exist in absolute values, still within broad statistical ranges, trends and conclusions drawn from the effects of treatment are the same.

LNCaP PCa cells were imaged first as controls at all three wavelengths for three different FOVs, treated on stage with doxorubicin and reimaged at identical FOVs at 20 min intervals for 2 h (Fig. 9). Either excitation approach would have reached the same conclusion that the cellular REDOX state or FLIRR increases after doxorubicin treatment. In our previous publications, we proceeded to perform additional, more nuanced segmented cell analysis, identifying subpopulations, based on treatment response, deemed unnecessary for this proof-of-principle technology paper.

**Fig. 10:**
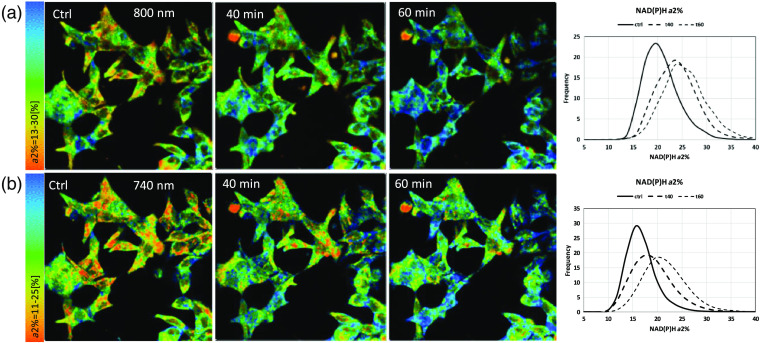
NAD(P)H-a2% images at 800-nm versus 740-nm excitation in LNCaP PCa cells. (a) 800-nm excitation for NAD(P)H control and 40 min, 60 min after doxorubicin treatment; histogram displays the frequency distribution of the enzyme-bound fraction of NAD(P)H. (b) NAD(P)H-a2% at 740-nm excitation, and the respective frequency histogram.

**Fig. 11:**
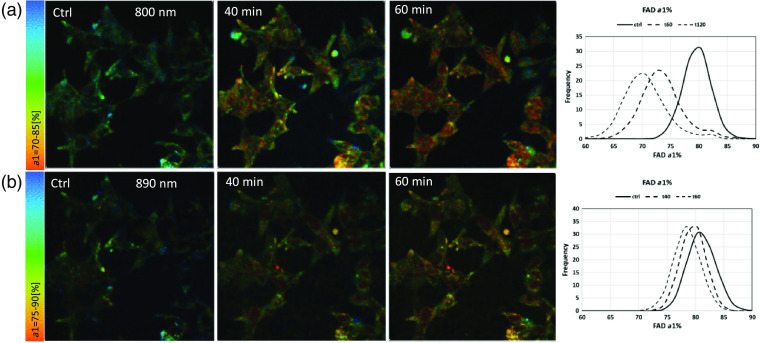
FAD-a1% images at 800-nm versus 890-nm excitation in LNCaP PCa cells. (a) 800-nm excitation for FAD control and 40 min, 60 min after doxorubicin treatment; histogram displays the frequency distribution of quenched fraction of FAD. (b) FAD-a1% at 890-nm excitation, and the respective frequency histogram.

**Fig. 12:**
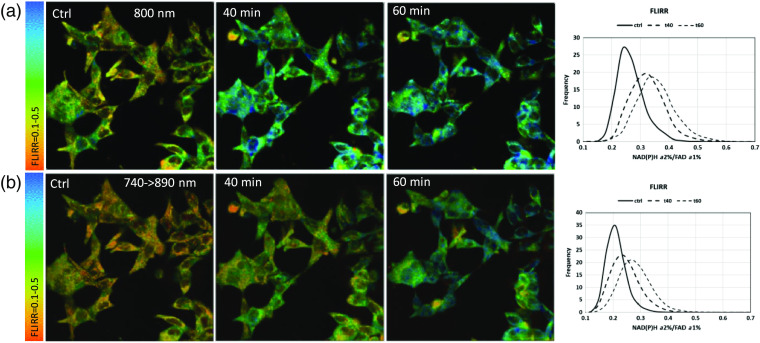
FLIRR images at 800-nm versus traditional excitation in LNCaP PCa cells. (a) 800-nm excitation for FLIRR control and 40 min, 60 min after doxorubicin treatment; histogram displays the frequency distribution of FLIRR. (b) FLIRR at 740-nm excitation for NAD(P)H and 890 for FAD, and the respective frequency histogram. Better FLIRR signal-to-noise image for single excitation 800 nm compared to two excitation wavelengths.

Figures 10–12 show representative images for data in Fig. 9 at control, 40 min, and 60 min. As we have observed in previous publications, this representative example of LNCaP cells exhibits the heterogeneous nature of individual cells at control and after treatment and hence, treatment response, with rising enzyme-bound NAD(P)H-a2%, a component of the REDOX marker FLIRR. In Fig. 11, FAD-a1% shows the previously published decline after treatment, more clearly seen at the 800-nm excitation. Figure 12 confirms the rising FLIRR ratio, also published previously,^34^^,^^35^ demonstrating that the 800-nm excitation produces the same results as the traditional excitation wavelengths.

### 3.7 FLIM Parameters of NAD(P)H and FAD at Different Excitation Wavelengths in Human Prostate Cancer Tissue Section

**Fig. 13:**
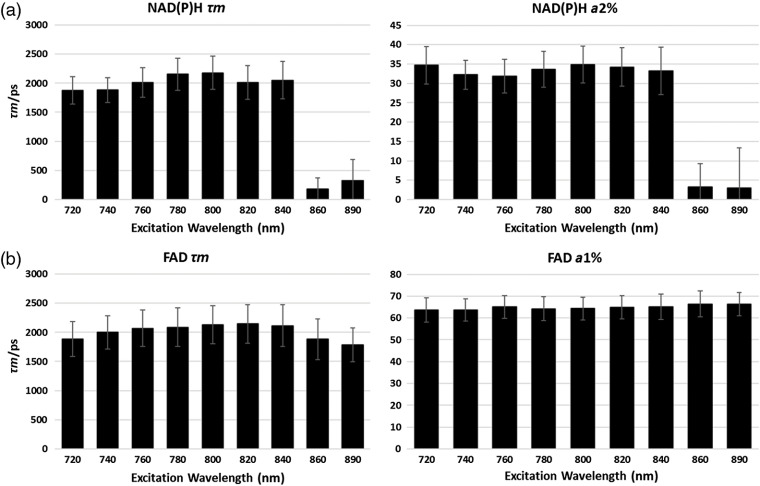
Fluorescence lifetime parameter results at different excitation wavelengths in the FAD channel for human PCa tissue section. (a) Mean data for NAD(P)H-τm and a2%; the low means at 860 and 890 nm are signals from second-harmonic generation. (b) Mean data for FAD-τm and a1%.

Human PCa tissue section demonstrates the successful application of the single wavelength excitation in tissues. Imaging was conducted at identical assay conditions as the AA and LNCaP cell lines to show that also here, the single 800-nm excitation wavelength compares well within statistical ranges with traditional approaches. The very low values for NAD(P)H at 860- and 890-nm excitation are as a result of second-harmonic generation, which is avoided by choosing 800 nm (Fig. 13).

## 4 Discussion and Conclusions

Fluorescence lifetime imaging has evolved rapidly in the last decade with the availability of new advanced instruments, software, and analysis methods and has become part of the imaging “tool chest” for scientific exploration. Challenges remain concerning image acquisition times, image processing, and analysis. Imaging the autofluorescent coenzymes NAD(P)H and FAD as a non-invasive method in the context of metabolism is increasingly being applied. Traditional FLIM assays involve sequential image acquisition, one for NAD(P)H at 700- to 740-nm excitation range and FAD at 890- to 900-nm range. The latter, which actually has a peak absorption at 760 nm, is moved to a higher wavelength to avoid exciting NAD(P)H. Sequential imaging additionally poses potential issues of motion artifacts and temporal resolution.

The idea of using a single-excitation wavelength for NAD(P)H and FAD is an obvious one and has been tried before,^53^ but not apparently in depth as demonstrated in this paper. These publications have used 800 nm without completely justifying this choice by carefully examining alternative wavelengths, particularly the NADH-SBT into the FAD channel was not addressed. The latest publication used a custom-built multilaser system to excite NAD(P)H and FAD simultaneously at high laser power levels.^52^ Our paper describes instead a method to use commercially available standard FLIM systems at standard laser power with one laser excitation.

In this paper, we have methodically moved from single aqueous solutions to mixed “calibration” solutions (approximating cellular conditions), followed by applying the same excitation wavelength ranges to three different cancer cell lines and a PCa tissue section, we finally decided on the 800-nm excitation wavelength, offering the most optimal combination. On the way, we tested optimal combinations of laser power and image acquisition time and achieved our objective by virtually eliminating any SBT from NAD(P)H into the FAD channel and back-SBT from FAD into the NAD(P)H channel. We had to compromise of accepting lower photon counts for NAD(P)H, however, quite sufficient for accurate lifetime fitting, with the associated benefit of increasing the photon count for FAD at a lower laser power than needed for the common 890-nm excitation. We finally applied the new assay to a doxorubicin treatment time-series of LNCaP PCa cells (Figs. 9–12) and showed comparable results to the sequential excitation 740/890 nm protocol and previously published data.^34^^,^^35^ For cells with a very different NAD(P)H and FAD concentrations, the single 800-nm excitation may not be optimal. To search for a more suitable single wavelength, the same method as presented in this paper can be used to find an alternative wavelength.

While there are some absolute differences in lifetime parameters at different excitation wavelengths, they are mostly in statistically acceptable limits. Publications in the FLIM field show that a narrow range of MP wavelengths are common [700 to 740 for NAD(P)H, 850 to 900 nm for FAD to avoid exciting NAD(P)H], presumably as a result of optimized instrument configurations. Considering environmental variables, absolute data levels are usually not as significant as changes occurring after some pharmacological or other intervention, which can be quantitatively demonstrated.

Apart from the simplified image acquisition protocol, there is a substantial reduction in the lifetime fitting and subsequent analysis process. Our method could be used for other NAD(P)H studies, for example for NADH and NADPH separation.^56^ This could also be a major advantage, when this assay is finally transferred to clinical applications in the cancer or other fields. The FLIM assay—as we have shown^33^—can predict optimal drug choices and their effects *in vitro* in a matter of hours versus days or weeks in patients. This translational step depends on the availability of biopsies in the case of PCa or acute myeloid leukemia patient serum samples (or equivalent cell lines with diagnosed, known mutations), which is in the realm of possibilities.
